# How social media sharing drives consumption intention: the role of social media envy and social comparison orientation

**DOI:** 10.1186/s40359-024-01627-7

**Published:** 2024-03-15

**Authors:** Dege Liu, Bin He, Ruan Feng, Xiaojun Huang, Gaoqiang Liu

**Affiliations:** 1 School of Management, Guangzhou University, No. 230 Wai Huan Xi Road, Guangzhou Higher Education Mega Center, 510006 Guangzhou, People’s Republic of China; 2 School of Law, Guangdong University of Finance & Economics, No. 21 Luntou Road, Haizhu District, 510320 Guangzhou, People’s Republic of China

**Keywords:** Social media sharing, Purchase intention, User-generated content, Social media envy

## Abstract

**Background:**

Social media benign envy, an upward comparison-based and painful emotions associated with the motivation to improve oneself, has attracted increasing attention from researchers due to its ubiquitous and significant impact on social network users’ intentions and behavior. However, the results of previous studies on whether material or experiential consumption is more likely to cause social media envy (treated as a single construct) have been inconsistent, and there is a lack of research on what triggers social media users to experience more intense benign envy and thus inspiring their consumption intentions. The purpose of this study is to investigate how the type and luxuriousness of shared consumption and viewer’s social comparison orientation jointly affect social media users’ consumption intentions through benign envy.

**Methods:**

A 2 (type of consumption sharing: experiential vs. material) × 2 (luxuriousness of consumption sharing: luxury vs. non-luxury) × 2 (social comparison orientation: high vs. low) mixed-design experiment was conducted to test theoretical model with data from 544 undergraduates in China. SPSS 26.0 and the Process macro were used to test the model.

**Results:**

The results revealed that luxury experiential consumption information shared on social media triggered more benign envy compared with other types of shared consumption information. When social media users shared non-luxury consumption, experiential consumption was more likely to inspire benign envy among users with high social comparison orientation than material consumption. However, when luxury consumption was shared, benign envy acted as a mediator between purchase type and participants’ purchase intention regardless of whether participants’ social comparison orientation was high or low.

**Conclusion:**

This study revealed that whether and how social comparison orientation of social media users who read the shared content influences the mechanism by which the type of consumption sharing on social media affects social media users’ consumption intentions through benign envy as a mediator is dependent on the luxuriousness of the shared consumption. The findings not only provide new insights for researchers to better understand social media envy and the underlying psychological mechanism for social media readers’ consumption intention, but also have practical implications for practitioners.

**Supplementary Information:**

The online version contains supplementary material available at 10.1186/s40359-024-01627-7.

## Introduction

Owing to the growth and popularity of the internet, social media (e.g., Facebook, Weibo, and Instagram) has become an increasingly indispensable medium of information and communication. Surveys have shown that people spend increasing amounts of time on social networks. In 2022, internet users worldwide spent an average of 147 min a day on social networks, up from 145 min in 2021 and the highest since 2012 [1]. People use social media to communicate with friends, express themselves, share their experiences and opinions, post comments, and browse information shared by others (such as viewing their activities) [2]. Several studies have suggested that social comparison based on traditional offline scenarios also occurs in the social media environment [3]. Social media users can easily and continuously access a large amount of information about others presenting their “good selves” and thus have many opportunities for social comparison [3, 4].

Social media envy, a painful emotion, arises when social media users engage in unfavorable upward social comparison due to their desire to possess but lack the possessions and life experiences shared in the social network by others (e.g., friends, social media influencers) [5, 6]. It has attracted increasing attention from researchers owing to its prevalent and significant impact on social media users’ consumption intention and behaviors [7, 8]. Research has shown that social media envy can have both positive and negative impacts. Whether social media envy leads to positive or negative consequences depends on the nature of the envy: malicious or benign [7, 8]. Social media benign and malicious envy are both upward comparison-based and painful emotions that can invoke different action tendencies and fulfill different functions [9]. While malicious envy causes hostility intention towards to the envied person, and decrease IT use intentions [8, 10], benign envy stimulates consumer desire for the coveted processions of others shared on social networks [11, 12]. Therefore, while curbing the negative consequences of social media malicious envy, it is vital to understand the triggers of benign envy and its constructive impacts on social media, and to apply its potential to bring about positive outcomes.

Although prior studies have provided preliminary evidence for our understanding of social media benign envy, a significant gap remains in the literature on what causes social media users to experience more intense benign envy (and how), and thus eliciting a range of different coping strategies. First, most previous studies that have treated social media envy as a single construct [13] may not provide an unequivocal answer on how to untangle the positive and negative effects of social media envy. To fully leverage the constructive impacts of social media envy while limiting its detrimental influences, it is crucial to understand how social media benign envy is elicited through which processes. Second, while some studies did not find a significant relationship between material and experiential consumption and social media envy [10], other research found that experiential consumption is more likely to trigger social media envy [14]. However, there has been little research paying attention to the moderating factors to resolve these differences. Third, although most prior social media envy research were conducted based on social comparison theory [7], little was known about the significant role of social comparison orientation (SCO) in how social media benign envy is triggered and thus elicit focal consumers’ envy-reducing responses: consumption intention.

We argue that the occurrence of social media benign envy can differ according to the type and luxuriousness of consumption shared by users on social media and the readers’ SCO. To fill the gap, this study aims to explore how the type and luxuriousness of shared consumption and viewer’s SCO jointly affect social media users’ consumption intentions through benign envy, and investigate the underlying mechanism for social media readers’ consumption intention. Specifically, based on social comparison theory, this study proposes and test a theoretical model to examine how the impact of the type and luxuriousness of shared consumption on viewer’s consumption intention through social media benign envy differs in terms of viewer’s SCO. By doing so, this study provides valuable new insights into how social media sharing can drive consumption intention through social media envy.

## Theoretical basis and hypotheses

### Social comparison in social media

According to social comparison theory, individuals have an intrinsic motivation to evaluate their abilities, opinions, and status, which leads to a constant comparison with others consciously or unconsciously [15], even in the absence of objective external standards for comparison [16]. Whenever individuals are confronted with information about others (e.g., groups, friends, family, classmates, and media-related entities), they relate this information to themselves [17, 18]. Social comparison satisfies the self-evaluation, self-enhancement, and self-improvement motivational needs of them by engaging in upward, downward, or parallel comparisons [19, 20]. It is a fundamental and universal psychological mechanism that affect individuals’ judgments, emotions, attitudes, and behaviors [20, 21].

Social media provides a large amount of easily accessible information that can be used for upward social comparisons. To create a good public image among their friends on social networks and gain approval or positive feedback, social media users often proactively shape themselves on social media by sharing carefully chosen and modified pictures, and texts [22–24]. Such positive self-presentation shared on social media platform can convey numerous symbolic messages about a sharer’s identity, status, superiority, wealth, and achievements [25], and provide other social media users with continuous opportunities for upward social comparison [2, 26]. Through comparisons with others, social media users can determine whether their identity and status are superior, equal, or inferior to those of their “friends” and form evaluations of themselves [3, 27]. The results of such social comparisons can influence users’ cognitive and emotional experiences, behavioral intentions, and behaviors [7, 26, 28].

### Envy-inducing characteristics of shared content on social media

As a social phenomenon, envy can occur only in environments where social comparison information is available [29]. On social network sites, individuals encounter considerable information that portrays other people in a positive light and showcases a symbolic meaning (e.g., status, success, and happiness) beyond the content itself. Reading and processing such social information are prerequisites for individual social comparison and social media envy [7, 11, 29, 30]. When social media users become aware of their relative disadvantage in terms of achievements, possessions, or happiness compared to similar others in relevant domains and dimensions (and desire to possess them), they experience envy [29, 31, 32]. Some studies have provided evidence that viewing content with symbolic meaning in relevant domains can provoke envy. For example, Krasnova et al. [33] found that frequent browsing of such social media information could cause envy among viewers.

Although previous research has shown that, on social media, travel [34], leisure [33, 35], brands [6], appearance [36], and tangible objects (e.g., clothing, jewelry, cars, apartments, and mobile phones) [35, 37] with symbolic meaning can all provoke envy among social media users, we still lack a deep understanding of how different types of social media content and the level of meaning it embodies affects social media envy. We argue that regardless of the specific content shared by social media users, it can be described in terms of material, experience, luxury, and non-luxury characteristics.

Social media users often share their travel experiences or new purchase on social media, which are usually referred to as experiential and material purchase [38]. Experiential purchases are those made with the primary intention of acquiring a life experience, consisting of an event or series of events that one lives through. By contrast, material purchases are those made with the primary intention of acquiring a material good, which is a tangible object kept in one’s possession [38]. Luxury is regarded as a subjective, relative, and situational interpretation of life experiences or activities, and is often associated with a wide variety of goods and services, including fashion, clothes, vacations, cars, cruises, hotels, and wines [11, 39–43]. It is a multidimensional cognitive and emotional construct conveying the significance related to several physical (e.g., financial values) and psychological values (e.g., prudential, personal, and social values) that distinguish luxury products and services from their non-luxury counterparts [43].

Prior research has proved that experiential vs. material purchases distinctions are valuable for studying social media envy [37, 44]. However, luxury and non-luxury characteristics, which have received considerable attention in other fields (e.g., advertising psychology), received little attention from social media envy researchers, even though they are potentially powerful influencing factors of social media envy. We argue that social media envy is influenced not only by the material purchases and experiential purchases shared by others, but also by the interaction of material purchases and experiential purchases as well as the luxuriousness embodied in material purchases and experiential purchases.

### The interaction effect of experiential vs material purchases and luxuriousness of purchases on social media benign envy

Previous research has shown that experiential purchases have many advantages over material purchases in affecting individuals’ emotions, and this “experiential advantage” may be even more pronounced in social media context [45]. Researchers have identified several psychological processes that can explain experiential advantage. First, experiences are more central to one’s identity [38]. Experiential purchases can help individuals understand their inner and true selves, which are important reflections of their core selves [44, 46]. Therefore, compared to material purchases, experiential purchases have a closer relationship with one’s self-identity and are more capable of fulfilling identity-related functions [47]. Second, experiential purchases can bring more social value. Experiential purchases are more conducive to promoting communication and social relationships between individuals than material purchases because discussing experiences can facilitate self-expression, bring individuals closer and make them feel pleasant and accepted [44, 45]. Finally, experiential purchases provide individuals with thoughts and memories along with a sense of belonging and social connection; therefore, they are better able to satisfy individuals’ pursuit of a sense of meaning than material purchases, which is both a cognitive and emotional assessment of whether one’s life has purpose and value [48–50].

When social media users read experiential consumption information shared by others, they may realize that the carefully selected experience consumption shared by others conveys others’ superiority in relation to identity, social values, and meaningfulness. Such upward social comparison in related experiential consumption domain thus invokes social media envy. Therefore, we have sufficient reasons to believe that, compared to material consumption, the experiential consumption shared by social media users that conveys their identity, social value and meaningfulness can explain why it triggers the envy of information readers more easily. Given that the results of prior research have shown that most feelings of envy were essentially benign [10], and that the degree of social comparison caused by experiential purchase is lower than that caused by material purchase [51], it is reasonable to believe that reading about experiential purchases shared by others inspires more benign envy than reading about material purchases.

When considering the luxuriousness of material and experiential consumption, we argue that the experiential advantage effect is more prominent in arousing benign envy. As a highly subjective construct, luxury conveys symbolic meanings that satisfy personal and social needs. Individuals tend to consume luxury products and services to project a successful image and social status, and to create and reinforce a prestigious self-image and identity, fulfill intrinsic needs, and signal superiority of themselves [43, 52–54]. In line with this reasoning, compared with non-luxury material and experiential consumption, luxury ones offer higher quality and prices, higher-end image, better performance, exquisite design, and craftsmanship, and appear rarer, more unique and prestigious, and convey more symbolic and emotional/hedonic values [55, 56], endowing consumers with a sense of superiority over their peers, which is a prerequisite for upward social comparison [57]. Such luxury-associated superiority can easily stimulate upward social comparison and thereby inspire envy from others. On the contrary, non-luxury material and experiential consumption is less capable of conveying information with symbolic meanings, and therefore is also less likely to arouse envy from others. Empirical research also provided preliminary evidence that luxury experiential consumption, compared with non-luxury experiential consumption, is more likely to stimulate upward social comparison and thus trigger focal consumer’s benign envy [11]. Therefore, it is reasonable to believe that luxury experiential consumption shared on social media would trigger more benign envy from viewers than other types of consumption. Thus, we propose as follows:

H1: Compared with non-luxury experiential consumption, non-luxury material consumption, and luxury material consumption shared on social media, luxury experiential consumption shared by social media users evokes more benign envy among social media viewers.

### The role of social comparison orientation

SCO, as a personality trait, reflects the extent to which individuals compare themselves with others [58]. It captures individuals’ propensity to compare themselves with others in accomplishments, status, and experiences [59], and is normally distributed among all individuals. Compared with those who are low SCO, people with high SCO experience more uncertainty and instability in their self-concept and are more sensitive and interested in others’ thoughts, behaviors, status, and performance [58, 60]. High SCO individuals are more susceptible to social comparison information and are more likely to evaluate themselves frequently based on such information [61, 62]. Social networks provide abundant social comparison information and opportunities. Compared to low SCO individuals, high SCO individuals invest more time in social networks and engage in frequent social comparison [63–65]. Moreover, such social comparison often results in negative effects. Park and Baek’s research showed that individuals with a higher ability-based SCO deliberately chose to compare themselves with those better than themselves to promote self-improvement, which in turn stimulated their envy and reduced their happiness [66]. Park and Jang’s research on tourism suggests that participants with high SCO experience more envy than their counterparts [67].

As one of the most important personality traits, SCO can influence the social comparison process and individuals’ responses to social comparison information [61]. Comparisons based on sharing information on social media have different consequences for individuals with high and low SCO. For instance, Yang [68] investigated how Instagram activities influence users’ psychological well-being and found that Instagram interaction can significantly reduce the loneliness of individuals with low SCO. Vogel et al.’s [65] research suggested that participants with a high SCO reported lower self-esteem and more negative affect after viewing Facebook profiles of acquaintances. Overall, findings of previous research indicate that, compared to those with low SCO, individuals with high SCO are more inclined to make social comparisons and suffer more.

Based on the above analysis, we argue that SCO may have impact on social media benign envy jointly with the type and luxuriousness of shared consumption. Luxury products and services convey more superiority and symbolic meaning than non-luxury products and services, therefore the impact of SCO on the individuals’ envy might be attenuated by the effect of luxury consumption information. By contrast, the role of SCO in inspiring individuals’ envy is more prominent when social media users browse non-luxury materials and experiential consumption information. Specifically, when social media users browse luxury consumption information, both material and experiential consumption information affect their benign envy, regardless of whether their SCO is high or low. On the contrary, when social media users browse non-luxury consumption information, experiential consumption information triggers more benign envy among individuals with a high SCO, who are more involved in the social comparison process of material and experiential consumption information. In this process, owing to the experiential advantage effect, non-luxury experiential consumption can evoke more benign envy among individuals with a high SCO. Therefore, we propose the following hypothesis:

H2: When consumption shared on social media is non-luxury, viewers who are high SCO experience more benign envy when they view experiential (vs. material) consumption.

### The mediating role of social media benign envy

As a painful emotion result from upward social comparison, envy is an important factor influencing individual behavioral intentions and behaviors [69]. Research based on offline scenarios shows that individuals can reduce envy by narrowing the gap between themselves and the target of comparison. They can choose “self-enhancement” behavior by striving to achieve or surpass the level of envy target, or “destructive” behavior by bringing the envied person down to their own level or making them lose the envied object. The stronger the desire to reduce envy, the more likely they were to engage in these types of behaviors [69]. Which behavior individuals choose depends on the nature of the envy [70, 71].

Similar to envy triggered in offline context, research has found that social media envy inspired by consumption of social network information also leads to envy-reducing behavioral intentions and behaviors [10, 11]. However, unlike envy in offline context, most of the envious emotions are actually benign in the social media context, which motives enviers to reduces gap with comparison targets by enhancing themselves or trying to obtain the products or services they desire [10, 11]. Therefore, it is plausible to argue that benign envy plays an important mediating role between shared information consumption and coping strategies [11, 72]. Based on the aforementioned reasoning on how the type and luxuriousness of consumption shared on social media and SCO jointly elicit envy, we hypothesized the following:

H3: The joint impact of type and luxuriousness of consumption on purchase intention is conditionally mediated by benign envy. In other words, when non-luxury consumption information is shared, benign envy mediates the impact of experiential consumption on the purchase intention of social media viewers with a high SCO.

**Fig. 1:**
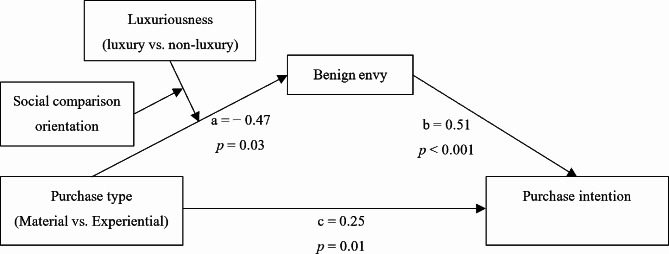
The proposed theoretical research model and path effects for the “users with high SCO” and “non-luxury consumption” condition

The theoretical model of the current study is shown in Fig. 1. As depicted, we propose a joint effect among the type of shared consumption, the luxuriousness it embodies, and SCO of the focal consumer on his or her consumption intention through social media benign envy. Specifically, the effect of the type of shared consumption on focal consumers’ benign envy depends not only on the luxuriousness it embodies (depicted in Hypothesis 1), but also on focal consumer’ SCO (depicted in Hypothesis 2). Furthermore, the joint effect of the type and luxuriousness of shared consumption on the focal consumers’ consumption intention is conditionally mediated by benign envy (depicted in Hypothesis 3).

## Method

### Design and stimuli

A 2 (type of consumption sharing: experiential vs. material) × 2 (luxuriousness of consumption sharing: luxury vs. non-luxury) × 2 (SCO: high vs. low) mixed-design experiment was conducted to test the hypotheses. While the trait SCO was measured within participants, the type and luxuriousness of consumption shared on social media were manipulated between participants using four experimental scenarios: luxurious experiential (a trip to Tokyo, Japan), non-luxurious experiential (a trip to Changlong, China), luxurious material (Gucci shoes), and non-luxurious material (Adidas shoes) consumption.

To manipulate the luxuriousness of material consumption, we chose Gucci (luxury) and Adidas shoes (non-luxury) as material comparison objects. The annual report on the most valuable and strongest apparel brands for 2022 showed that Gucci was the third most valuable luxury brand globally, with high brand value and consumer recognition and an opulent image that symbolized luxury. Conversely, Adidas had a lower brand value than Gucci. While it emphasized innovation and sustainability that aligned with contemporary values, with its relatively affordable prices, it was accepted by many people and forms a favorable brand image. Therefore, we used Adidas to represent non-luxury consumption [73]^1^.

Drawing on a method used in research [11, 37], we used social media posts including three components (destination, picture, and text) to manipulate the type and luxuriousness of consumption shared on social media. To manipulate the luxuriousness of experiential consumption, we chose scenic spots in Japan with solitary views (luxury condition) and Changlong Amusement Park in China with high tourist density (non-luxury condition) as the destinations for the stimulus materials. Furthermore, we used textual descriptions that matched the destination pictures to reinforce the difference between luxury and non-luxury experiential consumption. In the luxury condition, we used words such as “fantasy,” “taste,” and “luxurious,” whereas in the non-luxury condition, we used words such as “nice,” “irritating,” and “delicious.” Shoes of similar styles and types were selected to manipulate the luxury nature of material consumption. We chose more luxurious Gucci shoes and less expensive Adidas shoes to represent luxury and non-luxury material consumption, respectively. In order to highlight the features of luxury and non-luxury material consumption, we used shoe pictures that displayed their brand logo, design language, and text descriptions that matched the pictures to further enhance their differences. In the luxury condition, we used words such as “delicate,” “luxurious,” and “upscale,” whereas in the non-luxury condition we used words such as “nice,” “comfortable,” and “soft” (see Appendix for the stimuli).

### Participants and procedure

We recruited the participants from two universities located in Guangxi and Guangdong Provinces who were active users of social media. First, after being informed of the purpose of the experiment and confirming their consent, participants were asked to fill in demographic information (including gender, age, education), specify frequency of browsing Weibo, and fill out SCO Scale questionnaire. Subsequently, they were randomly assigned to one of four hypothetical scenarios with instructions to immerse themselves in the material context: browsing Weibo during their leisure time after a busy day, where they encountered a post from one of their Weibo friends. Upon completion of reading the materials, they were requested to answer two experimental manipulation check questions and scales measuring our focused variables. After completing the experiment, each participant received a monetary reward of 5 RMB (approximately USD 0.80).

Data were obtained from 611 participants. After checking their responses, we excluded nine participants who did not use Weibo, and 45 who did not choose the required options (“strongly agree” or “strongly disagree”) for validity check items. In addition, we excluded 13 responses due to missing values or extreme multivariate outliers, yielding a final sample of 544 valid responses, of which 136 were from male respondents (25%) and 408 from female respondents (75%). The mean age was 21.45 (*SD* = 3.35). Of the participants, 92.50% had a bachelor’s degree. Regarding Weibo browsing frequency, 13.79% browsed less than once a month, 11% browsed 1-3 times a month, 9.40% browsed once a week, 21.70% browsed several times a week, 11% browsed once a day, 21% browsed several times a day, and 12.10% always browsed Weibo.

### Measurement

Unless otherwise specified, all variables are measured using a 7-point Likert scale ranging from “completely disagree (1)” to “completely agree (7)”.

#### Purchase intention

We measured purchase intention with a four-item scale adopted from Dodds, Monroe and Grewal’s [74] and Gefen and Straub’s [75] studies. A sample item is “The probability that I would consider buying the product is very high”.

#### Benign and malicious envy

We assessed benign and malicious envy with a 10-item scale by van de Ven, Zeelenberg [69]. Sample items for benign envy include “The person’s success inspires me to do better” and “I will work hard because I hope to achieve similar success”. Sample items for malicious envy include “I feel annoyed when the person’s success affects me” and “I would be happy if the person failed”.

#### SCO

We measured SCO with an 11-item scale by Gibbons and Buunk [58]. Sample items include “I often compare myself with others with respect to what I have accomplished in life” and “If I want to learn more about something, I try to find out what others think about it”.

#### Control variables

Benign envy and malicious envy are both pain-driven emotions that fulfill different but related functions when the envier encountering an inferior upward comparison [9, 32]. Prior research has provided the evidence that malicious envy is significantly related to benign envy, and may influence the relations among our focused variables [9]. Therefore, to eliminate the potential influence of malicious envy on the relationships between the variables of interest, and help us better understand the positive nature of social media benign envy, we controlled for malicious envy.

We used two questions to test the validity of the manipulation. The first question asked participants to identify the type of consumption shared on social media (1 = material consumption, 2 = experience consumption). The second question required participants to rate the luxuriousness of consumption shared on social media using a 7-point Likert type scale ranging from “very unluxurious (1)” to “very luxurious (7)”.

## Results

### Descriptive statistics and manipulation check

Table 1 shows means, standard deviations, correlations and Cronbach’s α for the main variables.

Regarding to manipulation check, the results indicated that there was significant difference between the evaluation of the participants assigned to the material and experience purchase conditions (*M*_experience_ = 1.87, *M*_material_ = 1.42; *t* (542) = 12.39, *p* < 0.001). And, there was significant difference between the evaluation of the participants assigned to the luxury and non-luxury conditions (*M*_luxury_ = 5.43, *M*_non−luxury_ = 4.35; *t* (542) = 13.13, *p* < 0.001). Hence, the experimental manipulation was proved to be successful.

**Table 1:** Means, standard deviations, correlations and Cronbach’s α for the main variables

Variables	Mean	SD	1	2	3	4	5	Cronbach’s α
1. SOC	4.32	0.92						0.87
2. Benign envy	4.25	1.26	0.27^***^					0.94
3. Malicious envy	2.22	1.25	0.20^***^	0.14^***^				0.96
4. Purchase intention	4.41	1.28	0.27^***^	0.53^***^	0.07			0.94
5. Type	0.50	0.50	0.07	0.17^***^	− 0.14^**^	0.18^***^		
6. Luxuriousness	0.50	0.50	0.06	0.18^***^	0.15^***^	0.07	0.00	

Notes: *N* = 544; Type = the type of purchase shared on social media; Luxuriousness = the luxuriousness of the purchase shared on social media. *p<0.05; **p<0.01; ***p < 0.001 (two-tailed)

### Hypothesis tests

#### Testing for the effect of the type and luxuriousness of consumption sharing on benign envy

**Fig. 2:**
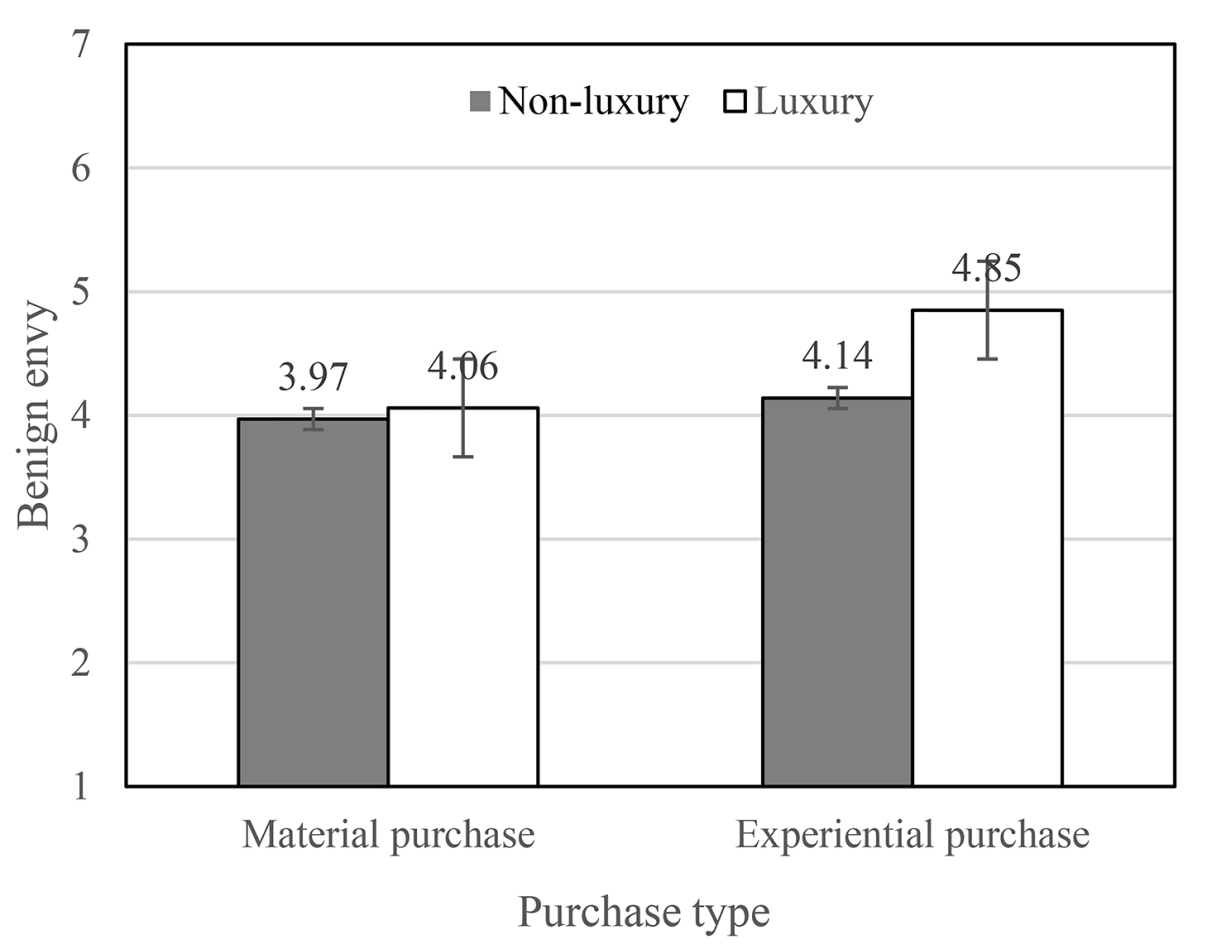
The interaction effect of the type and luxuriousness of purchases on social media benign envy

We used the two-way ANOVA to test Hypothesis 1. The results indicated that there were significant differences in the effects of consumption type (*F* (1,539) = 21.71, *p* < 0.001), luxuriousness of consumption (*F* (1,539) = 14.54, *p* < 0.001), and their interaction (*F* (1,539) = 9.07, *p* < 0.001) on benign envy (Table 2; Fig. 2). Specifically, on social media, luxury experiential consumption (*M* = 4.85) elicited higher levels of benign envy than non-luxury experiential consumption (*M* = 4.14), non-luxury material consumption (*M* = 3.97), and luxury material consumption (*M* = 4.06). As is shown in Table 3, when social media users shared experiential purchase, there is significant mean difference (M_*luxury*_ - M_*non−luxury*_ = 0.71, *p* < 0.001), whereas there is no significant mean difference (M_*luxury*_ - M_*non−luxury*_ = 0.09, *p* = 0.55) when social media users shared material purchase. Therefore, Hypothesis 1 was supported.

**Table 2:** Interaction effect of the type and luxuriousness of consumption information on benign envy

Source	Sum of squares	df	Mean square	F	***P***	Partial η²
Type	31.36	1	31.36	21.71	< 0.001	0.04
Luxuriousness	21.02	1	21.02	14.54	< 0.001	0.03
Type * Luxuriousness	13.10	1	13.10	9.07	0.003	0.02
Malicious envy	16.76	1	16.76	11.60	0.001	0.02
Error	778.78	539	1.45			
Total	10700.04	544				

Note: *N* = 554, Type = the type of purchase shared on social networks; Luxuriousness = the luxuriousness of the purchase shared on social networks

**Table 3:** Pairwise comparison of estimated marginal means

Consumption type	Mean Difference (M_luxury_ - M_non−luxury_)	SE	Sig.^b^	95% CI	
Material purchase	0.09	0.15	0.55	-0.20	0.37
Experiential purchase	0.71^*^	0.15	< 0.001	0.42	1.00

Notes: *N* = 554, Dependent Variable: Benign envy; Pairwise Comparisons was based on estimated marginal means; ^b^. Adjustment for multiple comparisons: Bonferroni. *p<0.05; **p<0.01; ***p < 0.001

### Testing for the moderating role of SCO

To test Hypothesis 2, we used a three-way interaction model proposed by Hayes (2013; Process Model 3) with the consumption-sharing type as the independent variable, the luxuriousness of the consumption and SCO as the moderating variables, and benign envy as the dependent variable. We conducted regression analyses using malicious envy as control variables. As shown in Table 4, in line with Hypothesis 2, there is a significant three-way interaction effect on benign envy (*b* = − 0.47, *p* = 0.03).

**Table 4:** Impact of type, luxuriousness, and SCO on benign envy

	Benign envy	
Variables	b	SE
Malicious envy	0.10	0.04
Type	0.45^***^	0.10
Luxuriousness	0.38^***^	0.10
SCO	0.28^***^	0.12
Type*Luxuriousness	0.54^**^	0.20
Type* SCO	0.31^**^	0.11
Luxuriousness* SCO	− 0.02	0.11
Type*Luxuriousness* SCO	− 0.47^*^	0.22

Notes: *N* = 544; Bootstrap sample size = 5000. Type = the type of purchase shared on social networks; Luxuriousness = the luxuriousness of the purchase shared on social networks; SCO = social comparison orientation. *p<0.05; **p<0.01; ***p < 0.001

**Fig. 3:**
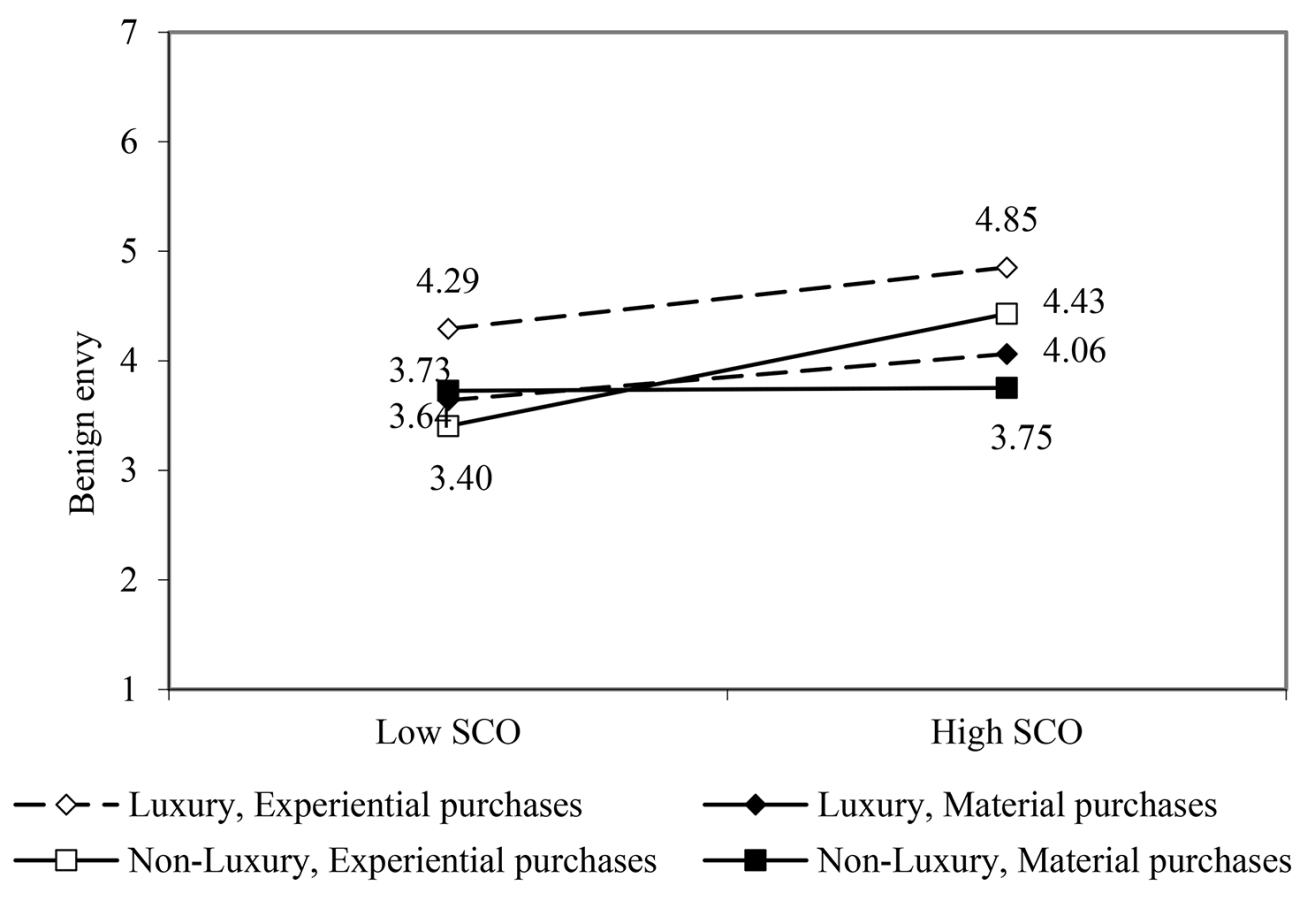
Simple slope tests for benign envy

As shown in Fig. 3, when shared consumption on social media was non-luxury, a significant interaction effect between consumption type and SCO on benign envy was found (*b* = 0.54, *p* < 0.001). Specifically, as shown in Table 5, when the SCO is high, experience consumption inspires more benign envy compared with material consumption (*b* = 0.68, *p* < 0.001). By contrast, when shared consumption on social media was luxury consumption, the results did not show a statistically significant interaction between consumption type and SCO in predicting benign envy (*b* = 0.08, *p* = 0.64). Hence, supporting Hypothesis 2.

**Table 5:** Simple slope test results for benign envy at different values of SCO and luxuriousness

	Low SCO (-1 SD)				High SCO (+ 1 SD)			
Variables	b	SE	95% CI - LL	95% CI - UL	b	SE	95% CI - LL	95% CI - UL
Non-luxury(0)	− 0.32	0.19	− 0.70	0.06	0.68^**^	0.20	0.28	1.08
Luxury(1)	0.65^**^	0.21	0.24	1.07	0.79^***^	0.20	0.40	1.18

Notes: *N* = 544; The type of purchase is independent variable, benign envy is dependent variable, SCO and luxuriousness are moderators; Bootstrap sample size = 5000

*p<0.05, **p<0.01; *p* < 0.001

### Testing for the mediating role of social media benign envy

To test Hypothesis 3, Hayes’s [76] moderated mediation model (Model 11) was employed. The results showed that there was an interaction effect among three predictive variables that significantly influenced benign envy (see Fig. 1, b = − 0.47, *p* = 0.03), and a significant effect of benign envy on purchase intention (*b* = 0.51, *p* < 0.001). However, as shown in Table 6, the 95% confidence interval for the index of moderated moderated mediation contains zero (Index = − 0.24, 95% CI = [− 0.52, 0.05]), so we cannot definitively conclude that SCO moderates the moderation of the indirect effect of purchase type by luxuriousness of purchase shared on social media. Hence, Hypothesis 3 was not supported.

**Table 6:** The index and effects for mediating role of benign envy using model 11

		Index	95% CI - LL	95% CI - UL
Moderated moderated mediation		− 0.24	-0.52	0.05
Conditional moderated mediation				
by SCO	Non-Luxury (0)	0.28	0.10	0.46
	Luxury (1)	0.04	− 0.17	0.27
		Conditional indirect effect	95% CI - LL	95% CI - UL
Low SCO (-1 SD)	Non-Luxury (0)	− 0.17	− 0.38	0.05
	Luxury (1)	0.34	0.05	0.63
High SCO (+ 1 SD)	Non-Luxury (0)	0.35	0.13	0.57
	Luxury (1)	0.41	0.20	0.66

Notes: *N* = 544; Percentile bootstrap CI based on 5000 bootstrap samples

Nevertheless, when we used PROCESS-macro for SPSS (Model 7) [76] to test the joint effect of purchase type and the luxuriousness of purchase shared on social media on purchase intention via benign envy, we found that there was an interaction effect between the purchase type and luxuriousness of purchase shared on social media that significantly influenced purchase intention through benign envy (Table 7, Index = 0.32, 95% CI = [0.10, 0.58]). Specifically, purchase type has a significant indirect effect on purchase intention through benign envy when the shared purchase was luxury (b = 0.41, 95% CI = [0.25, 0.61]). By contrast, such an effect was not significant for non-luxury shared purchase (b = 0.09, 95% CI = [− 0.07, 0.24]).

**Table 7:** The index and effects for mediating role of benign envy using model 7

	Index	95% CI - LL	95% CI - UL
Moderated mediation	0.32	0.10	0.58
	Conditional indirect effect	95% CI - LL	95% CI - UL
Pairwise contrasts between conditional indirect effects	0.32	0.10	0.58
Non-Luxury (0)	0.09	− 0.07	0.24
Luxury (1)	0.41	0.25	0.61

Notes: *N* = 544; Percentile bootstrap CI based on 5000 bootstrap samples

In sum, the findings demonstrated that the joint impact of type and luxuriousness of consumption on purchase intention through benign envy does not depend on the participants’ SCO. When consumption shared on social media was luxury, benign envy acted as a mediator between experiential purchase and participants’ purchase intention regardless of whether participants’ SCO was high or low.

## Discussion

The aim of the study was to explore how the characteristics of social media sharing can trigger social media envy and thus influence social media users’ purchase intentions. Using randomized mixed experiment, this study found that, compared with material and non-luxury experiential consumption, sharing luxury experiential consumption on social media triggered higher levels of benign envy among viewers. Moreover, we found that the type (experiential vs. material) and luxuriousness of consumption (luxury vs. non-luxury) shared on social media and viewers’ SCO jointly affected benign envy, and benign envy played a mediating role in the relationship between the joint effect of the purchase type and luxuriousness of purchase on participants’ purchase intention. Specifically, when consumption shared on social media was luxury, compared to material consumption, experience consumption triggers more benign envy, and thus enhancing participants’ purchase intention. By proposing and testing a conditionally mediating theoretical model, this study provides new insights for an understanding of the drivers and mechanisms of the emotional effects of social comparison on social network users, with several theoretical contributions and valuable managerial implications.

### Theoretical implications

First, this study provides new insights into research on experiential and material purchase by revealing their conditional effects on social media benign envy. As for whether material consumption or experiential consumption is more likely to cause envy among social media users, our findings demonstrated that luxury experiential consumption information stimulated more benign envy among social media viewers than other types of shared consumption information (H1), since luxury experiential consumption was shown to have an advantage compared to other types of consumptions. Furthermore, going beyond the previously discovered effect of experiential advantage on consumers themselves [7], this study provides further evidence that the experiential advantage effect plays a significant role in affecting the emotions of social media information viewers. Thus, this study contributes current social media envy research by reconciling inconsistent findings.

Second, this study sheds novel light on the role of SCO in provoking social media benign envy. Previous research has provided preliminary evidence for understanding whether browsing the information shared by other social media users (e.g., material and experiential consumption information) triggers envy [10, 11], and how individual characteristics (e.g., trait Self-esteem) and situational factors (e.g., luxuriousness of travel experience) jointly influence social media envy [11]. However, little relevant research has examined the role of social media viewers’ SCO in eliciting envy of themselves. Beyond previous research, we found that SCO does not always play its role in arousing envy among social media viewers. when the purchase shared by social media users are luxurious (whether it be material consumption or experiential consumption), social media readers’ SCO cannot play a significant role in eliciting envy. However, it is interesting that we found that when social media users share non-luxury consumption, there is a considerable difference in the impact of experiential and material consumption on benign envy among information viewers with low and high SCO (see Fig. 3). Viewers with a high SCO experience more benign envy inspired by non-luxury experiential consumption shared on social media, compared with non-luxury material consumption shared on social media (H2). Meanwhile, our findings open up an intriguing avenue for future research to explore: how to better leverage social media platforms to stimulate consumption intentions among those with low social comparison orientation.

Finally, this study provides valuable evidence for understanding the underlying psychological mechanism for social media readers’ consumption intention by testing how the type and luxuriousness of shared consumption and social media viewers’ SCO jointly effect their consumption intention through benign envy (H3). Our findings suggest that, although there was a significant interaction effect among the three predictive variables on benign envy, and benign envy had a significant effect on purchase intention, benign envy does not play a mediating role in the joint effect of these three predictive variables on purchase intention. Nevertheless, our findings uncovered a significant mediating role of benign envy in the relationship between experiential (vs. material) purchase and participants’ purchase intention when consumption shared on social media was luxury. These findings indicated that social comparison orientation does not play a role in the mechanism of how the type (experiential vs. material) and luxuriousness (luxury vs. non-luxury) of shared consumption jointly influences purchase intention through benign envy. Overall, our study addressed the research gaps in whether and how SCO and shared consumption information on social media interact to affect benign envy and purchase intention, thereby providing new insights for understanding the role of viewers’ personalities in the emotional mechanisms of social comparison on social network users. Moreover, our findings help advance the knowledge of peer influence mechanisms on social media from the perspective of social media information readers.

### Practical implications

First, this study provides a basis for social media platform providers to manage their platforms. While this study shows that browsing others’ shared consumption information can increase viewers’ purchase intention through arousing social media benign envy, this does not mean platform providers can unrestrictedly provide functions catering to users. In order to leverage the positive effects of social media posts, some users may include unrealistic luxury elements when describing non-luxury products/services, or conduct excessive image editing of shared products/services to highlight unrealistic luxury. While attracting users and maintaining the sustainable development of social media platforms, platform providers have an obligation to limit the potential negative impacts of such unrealistic information and guide objective posting through algorithm improvements. For example, platform providers could avoid providing functions that allow hiding information users do not want others to see, and/or limiting excessive image editing or beautification functions.

Second, our findings provide guidance for advertisers to optimize the effectiveness of their advertisements on social media. We found that luxury experiential consumption is more likely to elicit envy among social media users than other types of consumption, and that sharing non-luxury experiential consumption on social media is more likely to induce benign envy in social media users with high SCO, compared with sharing non-luxury material consumption. Thus, based on our research findings, advertisers can leverage the positive aspects of benign envy to stimulate users’ purchase motivation and improve advertising effectiveness on social media. In addition to including luxury elements in their advertising designs, advertisers should remember that when designing ads for goods and services, they should include experiential elements as much as possible, because luxury experiential consumption is more effective in eliciting benign envy and consumption intention in social media users. However, when designing ads targeting social media users with high social comparison orientation for non-luxury goods and services, they should be caution that including experiential elements in the ads may not yield intended results. Because the envy generated as a result may not necessarily translate into purchase intention.

Finally, the study findings can help firms increase their sales of products and services. Our research findings suggest that firms can stimulate consumers’ demand by encouraging users to share their comments regarding characteristics of purchased products or services (such as luxury experience) on social media platform, which can trigger other viewers’ benign envy and increase their purchase intention, ultimately increasing sales. It is important for firms to note that for non-luxury goods/services, their marketing activities that encourage customers to post comments about consumption experiences might not increase high SCO readers’ purchase intentions.

### Limitations and future research

First, the use of samples from China to test the research model might have limited the generalizability of our research results because we did not compare the differences between samples from different cultures in terms of the factors that triggered social media envy and their effects on consumer attitudes and behaviors. However, some research has indicated that cultural factors can influence social media envy and coping strategies [13]. Future researchers should gain a deeper understanding of social media envy using samples from countries with different cultural backgrounds and comparing the results among different social media platforms. Second, some participants’ idiosyncratic characteristics may influence the research results, such as the previous experiences, previously owned possessions, brand preference. Even though a randomized design was used in current study, we still can’t completely rule out their potential impact, future research could control for these idiosyncratic characteristics to eliminate their potential impact on research results. Third, owing to our research design, we could not draw conclusions about the causality between the variables based on our results. Future research should conduct in-depth analyses on related issues through a longitudinal design or experience-sampling methods. Finally, our study used experimental and survey-based methods to test the model, which might have limited the ability to acquire objective data from social media. Multi-method research can reduce errors that may be caused by a single method and provide more powerful evidence to draw reliable conclusions [8]. Given that social media data comes in a variety of formats (e.g., text, images, and video), future researchers can use methods such as text mining and big data analysis.

## Conclusion

Previous studies have conducted preliminary research on social comparison and envy in social network text; however, there remain numerous important unresolved issues [7, 30]. Our study revealed how shared information on social media could influence a social media users’ willingness to consume through benign envy. We found that sharing non-luxury experiential purchase on social media could stimulate higher levels of benign envy) among browsers with a higher SCO, and that luxury experiential (vs. material) purchase could evoke more benign envy among social media information browsers, regardless of their level of SCO, and thus enhancing their purchase intention.

Overall, our study provides new insights for a better understanding of the phenomenon of envy in the social network context, as well as holds practical implications for advertisers and businesses.

### Electronic supplementary material

Below is the link to the electronic supplementary material.

Supplementary Material 1

## Data Availability

The Data generated during the current study are available from the corresponding author on request.

## Footnotes

1 A pilot test with 54 participants was conducted to ensure the comparability of the type and luxury of consumption. The results indicated that Adidas shoes were perceived as more affordable than Gucci shoes (*M*_Adidas_ = 5.11, *M*_Gucci_ = 5.76, *t* = 2.41, *p* = 0.02), and that Guangzhou Changlong was considered more affordable than Japan (*M*_Changlong_ = 4.87, *M*_Japan_ = 5.52, *t* = 2.42, *p* = 0.02). Both were measured on a 7-point Likert-type scale, ranging from one to seven (1 = *very inexpensive* and 7 = *very expensive*).
