# Supplementary material for: How social media sharing drives consumption intention: the role of social media envy and social comparison orientation
Source: BMC Psychol. 2024 Mar 15;12:157. doi: 10.1186/s40359-024-01627-7 (PMC10943867; doi:10.1186/s40359-024-01627-7)
Supplement: Supplementary file 1 — Supplementary Material 1 [file 40359_2024_1627_MOESM1_ESM.pdf]

Appendix

Posts used in Study
Non-luxury material condition: Adidas

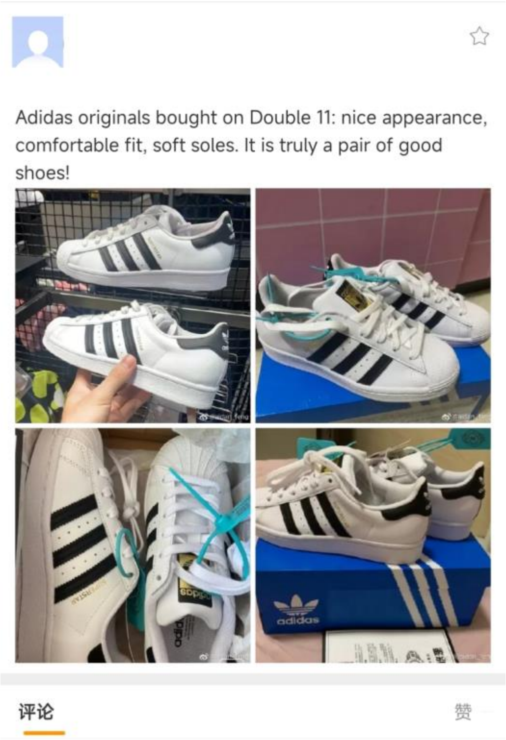

Luxury material condition: Gucci

Gucci shoes bought at a high price: delicate and luxurious appearance, upscale design. It is truly a pair of high-end shoes!

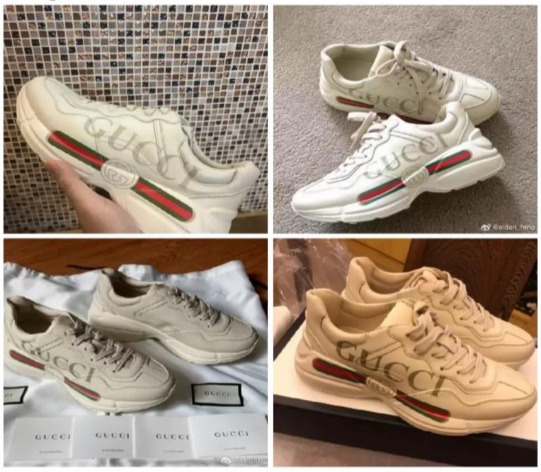

评论 赞

Non-luxury experiential condition: Chang long, Guangdong in China

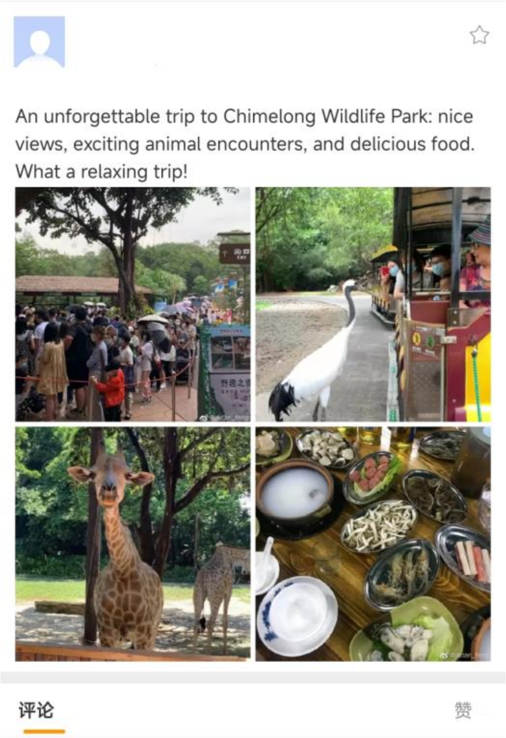

Luxury experiential condition: Tokyo, Japan

An unforgettable trip to Japan: fantastic scenery, the taste of sashimi and Kobe beef, and a luxurious hotel. What a relaxing trip!

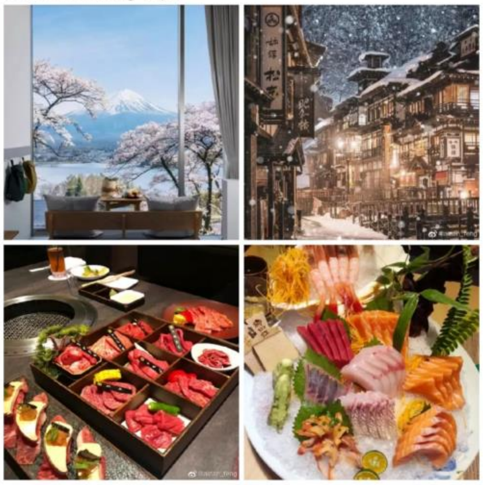

评论 赞
